# *De novo* assembly and characterization of *Camelina sativa* transcriptome by paired-end sequencing

**DOI:** 10.1186/1471-2164-14-146

**Published:** 2013-03-05

**Authors:** Chao Liang, Xuan Liu, Siu-Ming Yiu, Boon Leong Lim

**Affiliations:** 1 School of Biological Sciences, the University of Hong Kong, Pokfulam, Hong Kong, China; 2 Department of Computer Science, the University of Hong Kong, Pokfulam, Hong Kong, China

**Keywords:** Brassicaceae, *Camelina sativa*, Transcriptome, *de novo*, Paired-end sequencing, NBS-LRR

## Abstract

**Background:**

Biofuels extracted from the seeds of *Camelina sativa* have recently been used successfully as environmentally friendly jet-fuel to reduce greenhouse gas emissions. *Camelina sativa* is genetically very close to *Arabidopsis thaliana*, and both are members of the Brassicaceae. Although public databases are currently available for some members of the Brassicaceae, such as *A. thaliana*, *A. lyrata*, *Brassica napus*, *B. juncea* and *B. rapa*, there are no public Expressed Sequence Tags (EST) or genomic data for *Camelina sativa*. In this study, a high-throughput, large-scale RNA sequencing (RNA-seq) of the *Camelina sativa* transcriptome was carried out to generate a database that will be useful for further functional analyses.

**Results:**

Approximately 27 million clean “reads” filtered from raw reads by removal of adaptors, ambiguous reads and low-quality reads (2.42 gigabase pairs) were generated by Illumina paired-end RNA-seq technology. All of these clean reads were assembled *de novo* into 83,493 unigenes and 103,196 transcripts using SOAPdenovo and Trinity, respectively. The average length of the transcripts generated by Trinity was 697 bp (N50 = 976), which was longer than the average length of unigenes (319 bp, N50 = 346 bp). Nonetheless, the assembly generated by SOAPdenovo produced similar number of non-redundant hits (22,435) with that of Trinity (22,433) in BLASTN searches of the *Arabidopsis thaliana* CDS sequence database (TAIR). Four public databases, the Kyoto Encyclopedia of Genes and Genomes (KEGG), Swiss-prot, NCBI non-redundant protein (NR), and the Cluster of Orthologous Groups (COG), were used for unigene annotation; 67,791 of 83,493 unigenes (81.2%) were finally annotated with gene descriptions or conserved protein domains that were mapped to 25,329 non-redundant protein sequences. We mapped 27,042 of 83,493 unigenes (32.4%) to 119 KEGG metabolic pathways.

**Conclusions:**

This is the first report of a transcriptome database for *Camelina sativa,* an environmentally important member of the Brassicaceae. We showed that *C. savita* is closely related to *Arabidopsis* spp. and more distantly related to *Brassica* spp. Although the majority of annotated genes had high sequence identity to those of *A. thaliana*, a substantial proportion of disease-resistance genes (NBS-encoding LRR genes) were instead more closely similar to the genes of other Brassicaceae; these genes included *BrCN*, *BrCNL*, *BrNL*, *BrTN*, *BrTNL* in *B. rapa*. As plant genomes are under long-term selection pressure from environmental stressors, conservation of these disease-resistance genes in *C. sativa* and *B. rapa* genomes implies that they are exposed to the threats from closely-related pathogens in their natural habitats.

## Background

*Camelina sativa* is a dicotyledonous plant in the Family Brassicaceae. It is commonly known as *Camelina*, “gold-of-pleasure” or false flax. It has a growth cycle similar to that of *Arabidopsis*[1]. *Camelina* has been cultivated as a source of vegetable oil in Europe, central Asia and North America. The life cycle of *C. sativa* is relatively short, spanning approximately 100–120 days; thus, the species is very suitable for renewable-resource generation and as a spring or fall rotation crop [2]. *C. sativa* has 3,500 years of cultivation history. Although it is an ancient crop, *Camelina* cultivation has decreased gradually in modern times in relation to rapeseed [3]. The majority (80%) of fatty acids in *Camelina* oil are unsaturated; these are an important source of omega-3 fatty acids [4]. In *Camelina* seeds, polyunsaturated fatty acids constitute more than 50% of the total, and linolenic acid (18:3n-3) makes up about 35-40% of total fatty acids [3]. *Camelina* is recommended as a dietary supplement because of these benefits. In addition to its dietary use, *Camelina* oil has non-food applications, such as soaps, varnishes and biodiesel [5,6]. Production of this oil may solve the problem of limited feedstock availability for bio-diesel production. *Camelina sativa* would be useful as an alternative crop for biodiesel due to its low cost of production and high energy content. Because of the relatively high ester yield, alkyl esters from *Camelina* oil have been used as biodiesel [7]. *Camelina* oil has also been used directly as a fuel for diesel transport engines [6]. A further advantage of *Camelina* seed oil is that it produces less CO_2_ than traditional mineral oil products [8]. Moreover, *Camelina* is more drought-resistant and frost-tolerant than rapeseed and can thus be grown on land with little fertilizer or on land that is fallow. In comparison with other oilseed plants, *Camelina* is particularly competitive on highly saline soils [9]. The species is also well-known for its elevated resistance to insect pests and pathogens [10].

A number of plant transcriptomes have been deeply sequenced and subjected to further analysis over the last decade, particularly in model species of monocotyledons (*Oryza sativa*) and dicotyledons (*Arabidopsis*). These analyses provide valuable databases for non-model plant species [11,12]. However, there is no EST or genomic sequence currently available for *Camelina sativa* in the GenBank database. Transcriptomic sequence data for this low-cost oilseed plant will provide a valuable source of genomic information for practitioners of plant sciences. In the present study, we adopted Illumina paired-end sequencing to analyze the leaf transcriptome of *Camelina sativa*. In total, 2.42 gigabase pairs were obtained by deep sequencing; unigenes involved in most metabolic pathways were detected by our procedures. This is the first report of a transcriptome database for this oilseed plant. We provide a public dataset for genetic analysis and biological study of *Camelina sativa*.

## Results

### Sequencing and transcriptome assembly by SOAPdenovo

Total RNA was extracted from young and mature leaves of *Camelina sativa.* Poly A^+^ mRNA was obtained by passing total RNA through a column of beads conjugated with oligo (dT); the product was then fragmented into short sequences (200-700nt). The shortened mRNA was transcribed to cDNA by reverse transcriptase before sequencing. Clean “reads” were filtered from raw reads by removal of adaptors, ambiguous reads and low-quality reads. Approximate 27 million clean reads (2.42 Gbp) with a mean length of 90 bp were obtained (GenBank accession number: SRA057100). Two assembly methods were adopted and compared (Table 1). We used SOAPdenovo to assemble all high-quality clean reads into contigs (37.24 Mbp longer than 75 bp), which were assembled into scaffolds (32.72 Mbp longer than 100 bp) that were in turn assembled *de novo* into unigenes (26.65 Mbp longer than 100 bp). We generated 204,190 contigs (length ≥ 75 bp) with a mean length of 182 bp and an N50 of 194 bp (Table 2). The majority of contigs (175,262) were in the range 100 – 400 bp, which accounted for 71.9% of total contigs (227,194 total reads) (Additional file 1: Figure S1a). In order to assemble scaffolds, the contigs were connected with N to represent unknown sequences between two contiguous contigs. Details of scaffold total number, mean length and the N50 value are given in Table 2. Most of the scaffolds (118,708/129,539 = 89.5%) reads had gap length ratios of < 0.01 (Additional file 1: Figure S1b) and the most frequent lengths (91.6%) were between 100 and 500 nt (Additional file 1: Figure S1c). After assembling the scaffolds *de novo* with SOAPdenovo software, we filled scaffold gaps with the lowest number of Ns so that each scaffold could not be extended from either side. Resulting sequences were defined as unigenes. In total, 83,493 unigenes were generated with a mean unigene length of 319 bp and an N50 of 346 bp (Table 2) (GenBank accession number: GABO00000000). There were 10,877 unigenes with ≥ 500 bp, and 1,871 unigenes with ≥ 1000 bp; the majority of unigenes (87.0%) had 100 to 500 bp (Figure S1d). The frequency distributions of unigene lengths and ratios of gap length to size of assembled unigene are depicted in Figures S1d and Figure S1e, respectively. Among all unigenes, 99.87% (83,386 unigenes) had gap lengths that were < 5% of total length (Figure S1e). The random distribution of reads in assembled unigenes is presented in Figure S1f to display sequencing bias.

**Table 1:** Comparison of SOAPdenovo and Trinity assembly results

	**SOAPdenovo**	**Trinity**
Total length (nt)	26,651,285	71,935,591
Total number (n)	83,493	103,196
N50	346	976
Mean length (nt)	319	697
100–500 nt	72,616	53,377
500–1000 nt	9,006	27,983
1000–1500 nt	1,339	12,228
1500–2000 nt	354	5,623
≥ 2000 nt	178	3,985

**Table 2:** Summary details of sequences produced by SOAPdenovo assembly after Illumina sequencing

	**Sequences (n)**	**Base pairs (Mbp)**	**Mean length (bp)**	**N50 (bp)**
Clean reads	26,942,130	2,424.79	90	-
Contigs (≥ 75 bp)	204,190	37.24	182	194
Scaffolds (≥ 100 bp)	129,539	32.72	253	284
Total unigenes (≥ 100 bp)	83,493	26.65	319	346

### *De novo* transcriptome assembly by Trinity

To verify SOAPdenovo assembly results, we also made use of the Trinity assembly method, which assembles full-length transcripts without reference genomes [13]. In total 103,196 transcripts with a total length of 71,935,591 bp were generated (GenBank accession number: GABL00000000), significantly exceeding the output from SOAPdenovo (26,651,285 bp for 83,493 unigenes). The mean length and N50 obtained by Trinity assembly were also longer than those obtained by SOAPdenovo (Table 1).

To further verify the assembly results from both methods, we mapped both unigenes (SOAPdenovo) and transcripts (Trinity) to *Arabidopsis thaliana* (TAIR10_cds_20101214) CDS sequences using BLAT (Table 3). Parameters of BLAT were set as default (minMatch=2, minScore=30, minIdentity=90, maxGap=2, tileSize=11 and stepSize=11). Of 83,493 unigenes, 52,864 were successfully mapped to CDS in TAIR; 70,753 of 103,196 transcripts were also successfully mapped. Of the total CDS number in TAIR, 63.4% (22,435/35,386) and 61.4% (22,433/35,386) were mapped with sequences assembled by SOAPdenovo and Trinity, respectively. Among the mapped CDS in TAIR, 59.6% (21,084/35,386) were generated by both methods. Thus, similar mapping results were obtained from the two assembly methods when the *Arabidopsis thaliana* genome was taken as a reference genome. We adopted SOAPdenovo output for further analyses.

**Table 3:** Assembly results with TAIR 10.0 CDS using SOAPdenovo and Trinity software

	**SOAPdenovo**	**Trinity**
Number of unigenes/transcripts (n)	83,493	103,196
Number of unigenes/transcripts mapped to TAIR CDS (n)	52,864	70,753
Total number of CDS in TAIR (n)	35,386	35,386
Total number of mapped CDS in TAIR(n)	22,435	22,433
Total length of CDS in TAIR (bp)	43,546,300	43,546,300
Total length of mapped CDS in TAIR (bp)	31,264,828	31,145,430
Total length of unigenes/transcripts (bp)	26,651,285	71,935,591
Total length of mapped unigenes/transcripts(bp)	18,193,518	55,883,786
Total length of overlapping sequences (bp)	15,234,784	44,020,562
Percentage of mapped CDS number	22,435/35,386 = 63.4%	22,433/35,386 = 63.4%

### Annotation of predicted proteins in NR and Swiss-Prot databases

**Figure 1:**
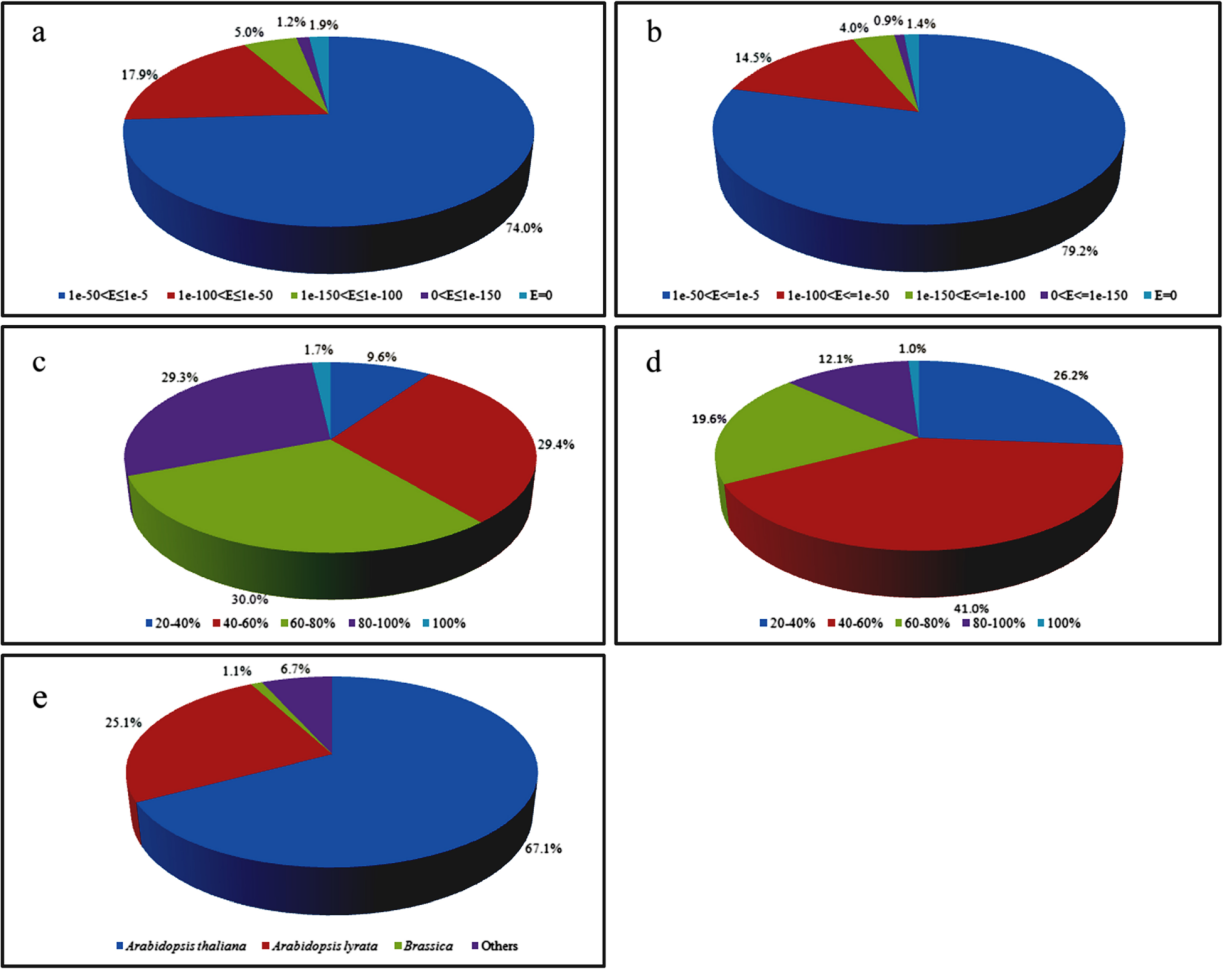
**Unigene homology searches against NR and Swiss-Prot databases.** (**a**) E-value proportional frequency distribution of BLAST hits against the NR database. (**b**) E-value proportional frequency distribution of BLAST hits against the Swiss-Prot database. **(c)** Proportional frequency distribution of unigene similarities against the NR database based on the best BLAST hits (E-value ≤ 1.0E-5). (**d**) Proportional frequency distribution of unigene similarities against the Swiss-Prot database based on the best BLAST hits (E-value ≤ 1.0 E-5). (**e**) Proportional homology distribution among other plant species based on the best BLAST hits against the NR database (E-value ≤ 1.0 E-5).

In total, 67,791 unigenes were annotated with 25,329 unique protein IDs. Most protein IDs (25,075 of 25,329) were annotated by the NR database. To annotate gene names and protein coding sequences among unigenes, we searched all six-frame translations of unigenes against the NR plant protein database in NCBI by running BLASTX with an E-value cut-off 1.0E-5. In total, 67,497 BLASTX hits (matches) were obtained, covering 80.8% of all unigenes (Table 4 and Additional file 2). Of these, 45,307 and 16,969 BLASTX hits were matched to *Arabidopsis thaliana* (TAIR 9) and *A. lyrata* proteins, respectively. Only 722 BLASTX hits were aligned to NR protein of *Brassica* spp. (Table 4). Thus, *Camelina sativa* was genetically more closely related to *Arabidopsis* than *Brassica*. The E-value frequency distribution of significant hits (E-value ≤ 1.0E-50) showed that 26% of the sequences shared strong homologies; the majority of matched sequences (74%) had E-values in the range 1.0E-50–1.0E-5 (Figure 1a). The translated amino acid sequences of unigenes were also closely similar to sequences from the NR database; nearly 90% of the BLASTX hits were in a similarity range between 40% and 100%. Only 9.6% of hits had similarity values less than 40% (Figure 1c). Homologies between different species are depicted in Figure 1e. Among hits, 67.1% matched to *Arabidopsis thaliana*, followed in sequence by *A. lyrata* (25.1%) and *Brassica* (1.1%) (Figure 1e).

**Table 4:** **Summary of *Camelina *unigene annotations from SOAPdenovo assembly**

	**Unigenes (n)**	**Annotations (n)**	**Functional classification**
All assembled unigenes	83,493	-	-
Gene annotations against *Arabidopsis thaliana* protein of NR	45,307	45,307	-
Gene annotations against *Arabidopsis lyrata* protein of NR	16,969	16,969	-
Gene annotation against *Brassica* protein of NR	722	722	-
Unique gene annotations against plant NR	67,497	67,497	-
Gene annotation against Swiss-Prot	40,804	40,804	-
Gene annotation against COG	14,190	14,190	25 categories
Gene annotation against KEGG	27,042	27,042	119 pathways
GO annotations for biological process	23,524	49,164	27 sub-categories
GO annotations for cellular component	25,885	37,439	9 sub-categories
GO annotations for molecular function	26,825	30,888	10 sub-categories
All annotated unigenes	67,791	-	-
Unigenes matching all four databases	11,685	-	-

We also matched protein coding sequences of unigenes with the protein database at Swiss-Prot using BLASTX; 40,804 of 83,493 unigenes (48.87%) returned hits at an E-value threshold of ≤ 1.0E-5 (Table 4). Most of the matched sequences (79.2%) had E-values between 1.0E-50 to 1.0E-5, and the remaining 20.8% had strong homologies with E-values of ≤1.0E-50 (Figure 1b). The similarity frequency distribution against Swiss-Prot was different from that obtained against the NR database; 86.9% of query sequences against Swiss-Prot had similarities between 20% and 80%; only 13.1% of sequences had strong homologies with >80% identity (Figure 1d).

### Conserved domain annotation and COG classification

**Figure 2:**
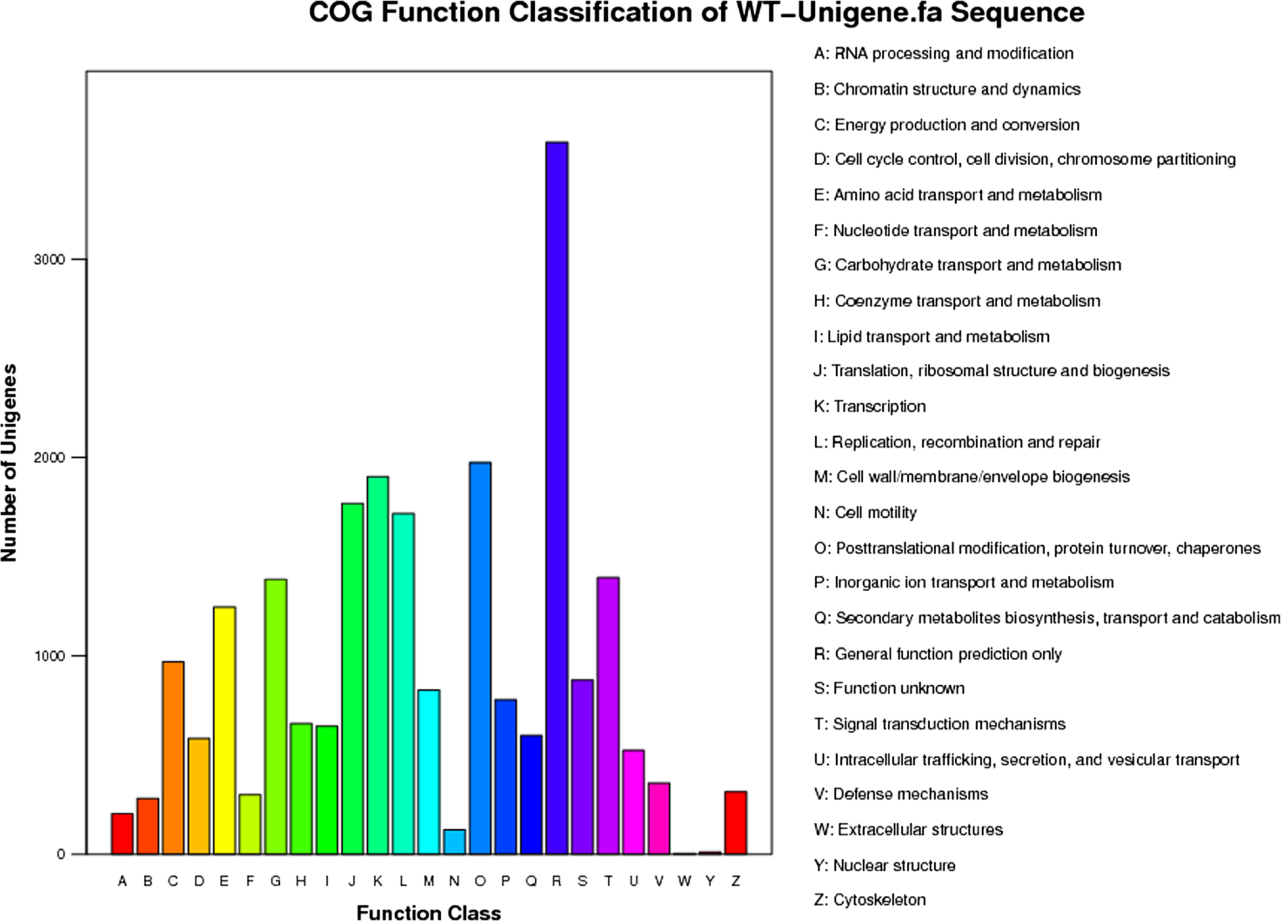
**COG functional classification of the *Camelina sativa *transcriptome.** Of 67,497 hits in the NR database, 14,190 unigenes with significant homologies in the COG database (E-value ≤ 1.0 E-5) were classified into 25 COG categories.

COG is a database built on phylogenetic relationships of protein sequences from 66 genomes, including bacteria, plants and animals. Individual proteins or paralogs from at least three lineages are categorized in each COG to represent an ancient conserved domain. Within the *Camelina sativa* unigene set, 14,190 were categorized (E-value ≤ 1.0E-5) in 25 functional COG clusters (Table 4, Figure 2). Thus, only a small proportion of the unigenes (17.0%) carried protein domains with annotation for COG categories. The five largest categories were: 1) general function predictions only (15.6%), 2) post-translational modification, protein turnover, chaperones (8.6%), 3) transcription (8.3%), 4) translation, ribosomal structure and biogenesis (7.7%), and 5) replication, recombination and repair (7.5%).

**Figure 3:**
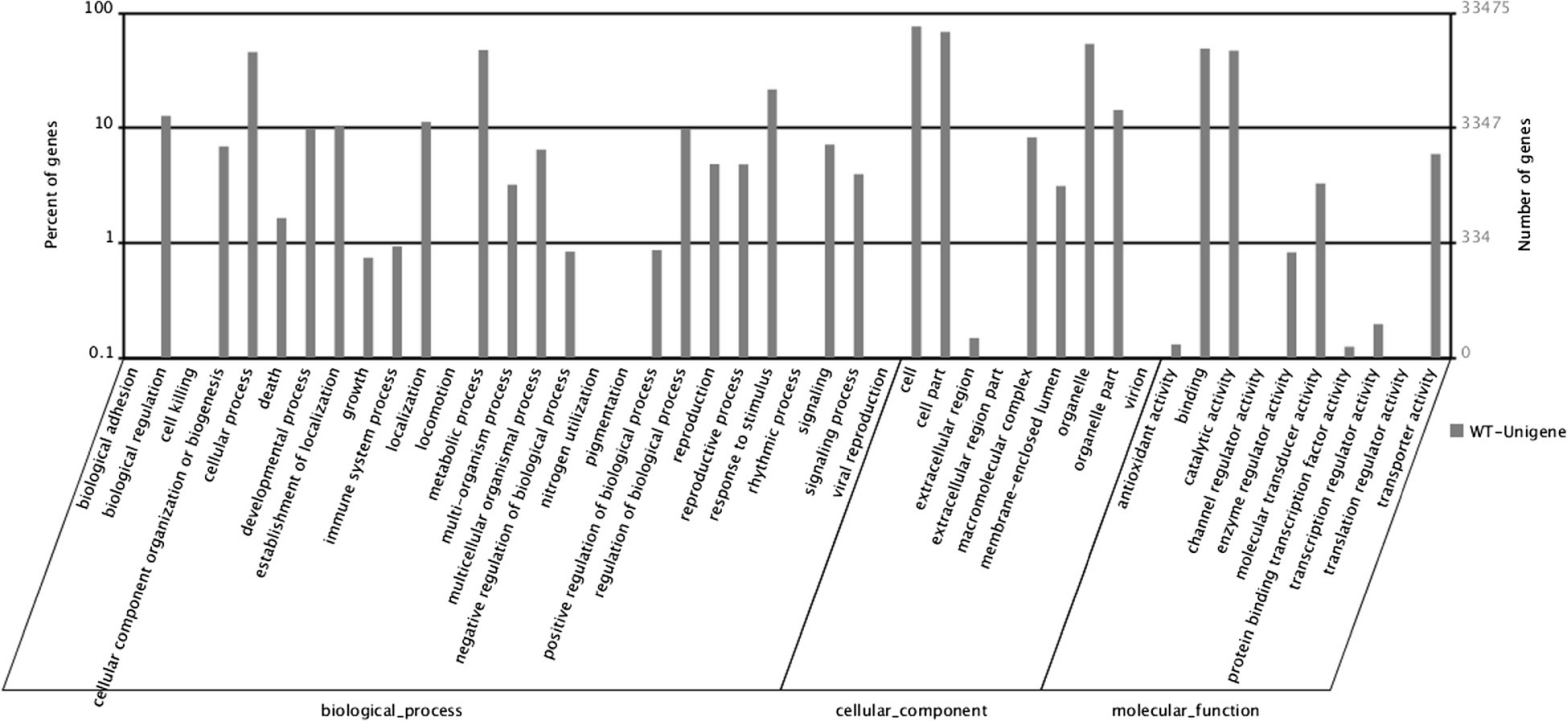
**Gene Ontology (GO) term assignment of *Camelina *unigenes.** Based on high-score BLASTX matches in the NR plant proteins database, *Camelina* unigenes were classified into three main GO categories and 46 sub-categories. The left y-axis indicates the percentage of a specific category of genes in each main category. The right y-axis indicates the number of genes in the same category. In total, we assigned 33,475 unigenes with BLASTX matches to known proteins.

### Gene ontology (GO) classification

**Figure 4:**
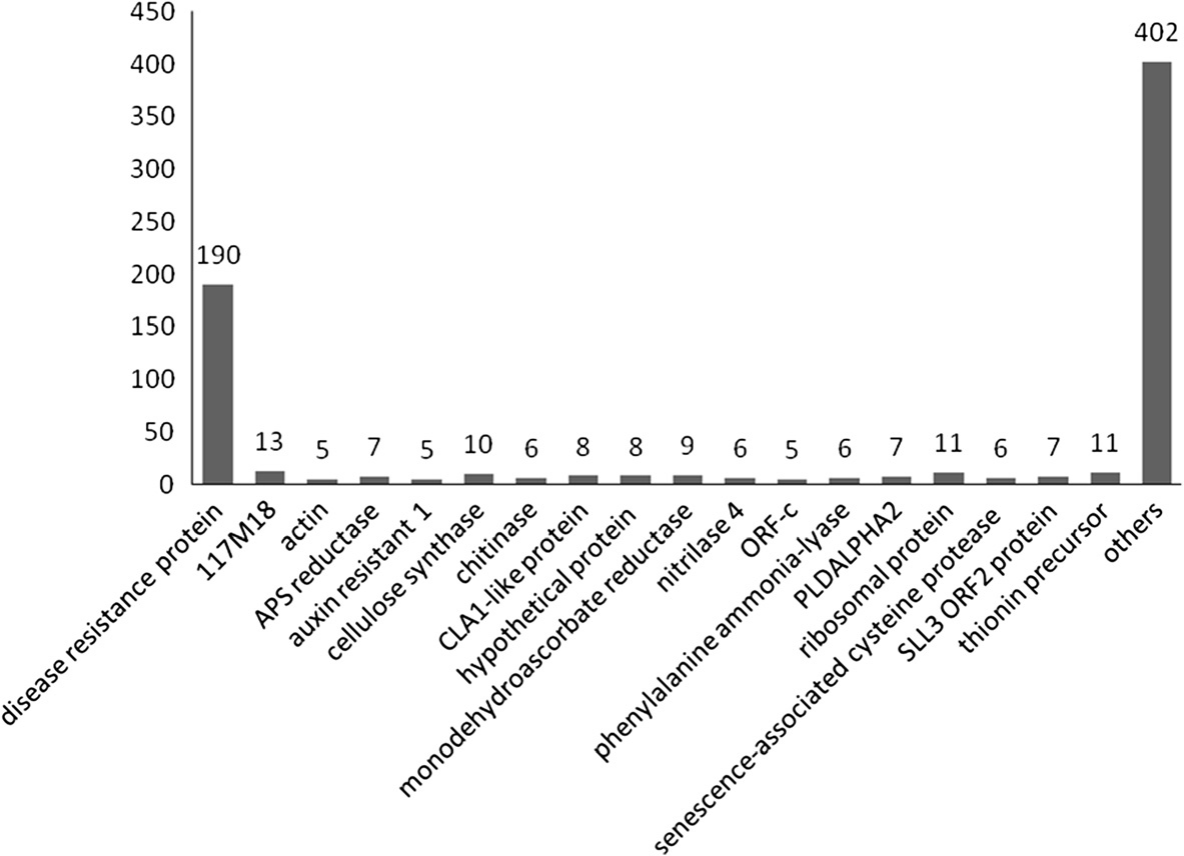
**Frequency distribution of unigenes that mapped to *Brassica *sequences in the NR database.**

To functionally categorize *Camelina sativa* unigenes, we assigned GO terms to each assembled unigene [14]. The *Camelina* unigenes were categorized in three main GO categories: biological process (23,524, 30.9%), cellular component (25,885, 34.0%) and molecular function (26,825, 35.2%). These GO terms were further subdivided into 46 sub-categories (Table 4 and Figure 3). In total, 33,475 unigenes were assigned to at least one of the GO categories of biological process, cellular component and molecular function (Additional file 1: Figure S2). The transcriptome of *Camelina sativa* was closely related to *Arabidopsis thaliana* sequences (Figure 1e). Only 1.1% of unigenes (722 unigenes) had higher homology with genes from *Brassica* spp*.* A substantial number (190) of these 722 unigenes were annotated as disease resistance proteins. They accounted for 26.3% of total unigenes annotated to *Brassica* sequences (Figure 4 and Additional file 3). After overlapping unigenes were filtered, 381 individual NR IDs matched *Brassica* sequences; among these, 50 NR IDs matched *Brassica* disease resistance proteins (Additional file 3).

### KEGG pathway mapping

Unigenes of *Camelina sativa* were mapped to KEGG pathways by using the translated amino acids to run BLASTX against the KEGG database. All returned hits with an E-value ≤ 1.0E-5 were annotated with Enzyme Commission (EC) numbers [15]. Using *Arabidopsis thaliana* as a reference for pathways analysis, we annotated and mapped 27,042 of 83,493 unigenes (32.4%) to EC numbers in 119 KEGG pathways (Figure S3 in Additional file 1 and Additional file 4). Metabolic pathways had the largest number of unigenes (6,469 members, 23.9%, ko01100), followed in sequence by secondary metabolite biosynthesis (3,600 members, 13.3%, ko01110), plant-pathogen interaction (2,004 members, 7.4%, ko04626), spliceosome (1,333 members, 4.9%, ko03040), protein processing in the endoplasmic reticulum (973 members, 3.6%, ko04141), and starch and sucrose metabolism (777 members, 2.9%, ko00500) (Additional file 4).

Overall, 67,791 unique sequence-based annotations had BLASTX scores with E-values ≤ 1.0E-5 in the NR, Swiss-Prot and KEGG databases (Additional file 1: Figure S3). Among this number, 11,685 unigenes had hits in all four public databases (NR, COG, Swiss-Prot and KEGG), with relatively well defined functional annotations (Table 4, Figure S3 in Additional file 1). These annotations provide a valuable resource for investigating specific processes, structures, functions, and pathways that will guide research on *Camelina sativa*.

### Protein coding sequence (CDS) prediction

Unigenes were firstly aligned by BLASTX (E-value ≤ 1.0E-5) to protein databases in the priority order NR, Swiss-Prot, KEGG, COG. Unigenes aligned to a high priority database were not aligned to databases of lower priority. The process ended when all alignments had been performed. The correct reading frame of the nucleotide sequences of unigenes (5’-3’ direction) was defined by the highest rank in the BLAST results, and the corresponding protein sequences were obtained from the standard codon table. Unigenes that could not be aligned to any database were scanned with ESTScan [16] to produce the nucleotide and amino acid sequences of the predicted region. In total, CDS of 67,739 unigenes were generated by BLASTX protein database searches described above. To evaluate the quality of the databases, we analyzed the ratio of gap lengths to the sizes of unigene CDS. Almost all of the unigenes had gap lengths that were < 5% of total length, accounting for 99.96% of total unigenes with CDS sequences (67,739 unigenes) (Additional file 1: Figure S4a). The majority of the unigene CDS (88.30%) had < 500 bp. Of 67,739 unigenes with CDS sequences, 7,923 had ≥ 500 bp and 1,340 had ≥ 1000 bp. The size frequency distributions of these unigene CDS and proteins are depicted in Figure S4b and S4c, respectively (Additional file 1). We scanned 1,042 CDS of unigenes that could not be aligned to any database by ESTScan. Of these, only two had gaps (Additional file 1: Figure S4d). The majority of the unigene CDS assigned by ESTScan (96.93%) were shorter than 500 bp (Additional file 1: Figure S4e); this was also the case for protein sequences obtained from ESTScan (Additional file 1: Figure S4f).

### Fatty acid pathways in *Camelina sativa* and *Arabidopsis thaliana*

Fatty acids are carboxylic acids with long hydrocarbon chains that are saturated or unsaturated. The carbon atoms per molecule vary from 10 to 30. In *Camelina* seeds, most of the fatty acids are unsaturated and the oil has high omega-3 fatty acid content (35-40%). Eggs from hens and milk from cows fed *Camelina* meal contain elevated contents of unsaturated fatty acids [17,18]. In our annotated *Camelina sativa* transcriptome unigene dataset, we identified key genes involved in fatty acid biosynthesis and unsaturated fatty acid biosynthesis (Additional file 5). *Camelina sativa* shares pathways similar to those of *Arabidopsis thaliana*[19]. However, not all gene members in the *Arabidopsis* genome were identified in the transcriptome dataset. For example, there are 7 homologous stearoyl-ACP desaturase genes (FAB2, DES1-6) in the genome of *Arabidopsis*[20], but only four of these can find highly homologous unigenes (E-values ≤ 1.0E-68) in the transcriptome dataset (data not shown). The other three members may not be expressed in leaves, or may be absent from the genome of *C. sativa*.

## Discussion

High-throughput sequencing is a superior technology for transcriptome analysis. Compared with traditional large-scale EST sequencing, it is less costly and more efficient. It is more suitable for use in non-model organisms whose genomic sequences are unknown. Prior to our work, there was no sequencing information on *Camelina sativa* in public databases. We used RNA-seq technology for transcriptome profiling to sequence RNA extracted from leaves. This *de novo* transcriptome sequencing technology has been used for many plants, including rice [21], maize [22], sesame [23], bamboo [24], poplar [25]. However, Illumina transcriptome analysis has been restricted previously to the sequencing of organisms for which reference genomes were available [26]. The Illumina sequence technology has improved recently, most notably through an expanded capability for *de novo* sequencing of relatively short reads and their assembly into unigenes by paired-end sequencing technology [27]. Paired-end sequencing not only increases the depth of sequencing, but also improves *de novo* assembly efficiency. Through this procedure, we obtained 2.42 G bp reads with 27.0 million clean reads. All of these reads were assembled into 83,493 unigenes by SOAPdenovo and 103,196 transcripts by Trinity. While the output was consistent with a previous study on the *Camellia sinensis* transcriptome [28], the hexaploid genome of *Camelina sativa* add complexity to the analysis [29]. When the assembled 83,493 unigenes and 103,196 transcript were mapped to Arabidopsis CDS (TAIR 10), 22,435 and 22,433 unique Arabidopsis CDS could be mapped, respectively (Table 3). Multiple unigenes could be mapped to the same CDS as they cover different regions of the CDS; and 2 or 3 unigenes could be mapped to the same CDS due to the polyploidy of Camelina. Six Camelina genes expressed in developing seeds were shown to have 3 haplotypes in ecotype CS32 [29]. To confirm that Camelina ecotype MT-5 is also a hexaploid, these 18 sequences were mapped to the clean reads and the unigenes/transcripts produced by SOAPdenovo and Trinity by BLASTN with perfect match and E-value ≤ 1.0E-5. Three out of the six genes could find 3 haplotypes from our clean reads database, confirming that ecotype MT-5 is also a hexaploid (data not shown). Some unigenes/transcripts are actually different haplotypes of the same gene. For example, three unigenes (Unigene5348_WT, Unigene30695_WT, Unigene34277_WT) were found to be the three haplotypes of the homologous gene of AT2G18040. This reflects the complexity of assembling polyploidy transcriptome. Currently no existing assembler can handle polyploidy well.

Transcriptome analysis indicates that *Camelina sativa* is closely related to *Arabidopsis thaliana* (Figure 1e). A particular example of this relatedness is the presence of *AtPAP2*(AT1G13900)-like (*CsPAP2*, unigenes 87, 6572, 7242, 25505, 46194, 52973 and 76943) and *AtPAP9*(AT2G03450)-like (*CsPAP9*, unigenes 129, 27542, 29608, 33278, 39915, 40207, 58111, 63089, 63449, 73151) sequences in the transcriptome of *Camelina sativa* (data not shown). CsPAP2 shares 91% amino acid sequence identity with AtPAP2; CsPAP9 shares 89% amino acid sequence identity with AtPAP9. In *Arabidopsis* and *Camelina*, two PAPs carry a C-terminal hydrophobic motif, but only one PAP-like sequence is known to carry this hydrophobic motif at the C-terminus in other plant genomes. For example, of 35 PAP-like genes in the soybean genome, only one PAP sequence (*GmPAP35*) carries this C-terminal motif [30]. Overexpression of AtPAP2 in *A. thaliana* produces fast-growth and high seed-yield phenotypes [31]. Overexpression of AtPAP2 in *Camelina sativa* also produces fast-growth and high seed-yield phenotypes [32]. It would be worthwhile investigating whether this effect is specific only to *A. thaliana* and *C. sativa*. Of particular interest would be identification of the physiological mechanism underlying this difference among plant species. Is this merely the result of a random gene duplication event during the evolution of *A. thaliana* and *C. sativa*, and what is the physiological function of the single PAP sequence with the specific C-terminal hydrophobic motif in other plant species?

Due to their close genetic relationship, *Camelina sativa* and *Arabidopsis thaliana* share similar KEGG pathways. We generated 119 KEGG pathways from the *C. sativa* transcriptome and compared them with pathways in *A. thaliana*. Some pathways in the two species were identical, including pathways involved in biosynthesis of unsaturated fatty acids, biotin metabolism, citrate cycle (TCA cycle), fatty acid elongation in mitochondria, glycosaminoglycan degradation, proteasome and protein export. Moreover, except in a few cases, most of the genes involved in these pathways were highly similar between the two species (Additional file 6).

While most of the unigenes had high sequence identity to *Arabidopsis* sequences (Figures 1e, f), a small proportion (1.1%) of defense-related unigenes were closely similar to *Brassica* sequences (Figure 4 and Table 4). We identified a non-trivial proportion of these as disease resistance genes. Plants have developed defense systems for protection against pathogen invasion. In plant genomes, the nucleotide-binding site (NBS)-encoding resistance genes belong to one of the largest gene families and account for approximately 2% of all genes. NBS-encoding genes of some monocotyledons and dicotyledons, including *Oryza sativa*[33], *Medicago truncatula*[34], *Brassica rapa*[35], and *Populus trichocarpa*[36], have been studied using model species as reference organisms. In *B. rapa*, 92 non-redundant NBS-encoding genes have been identified [35]. Of 722 *Camelina* unigenes that matched *Brassica* sequences, 190 were disease resistance genes (including BrCN, BrCNL, BrNL, BrTN, BrTNL and LRR) (Additional file 3). The conservation of these disease-resistance genes in *Camelina* over evolutionary time scales implies that *Camelina* and *B. rapa* have been exposed to the threats from closely-related pathogens.

Glucosinolates are secondary metabolites of the Brassicales that play important roles in plant defense. Most genes that encode glucosinolates biosynthesis in *Arabidopsis* have been clearly identified and may be used as reference loci for research on *Camelina* glucosinolate genes [37,38]. We screened all unigenes of *C. sativa* by matching them to the query sequence of *Arabidopsis thaliana* genes using BLASTX tools. Except for *AOP3, CYP79F2*, *MYB76* and *FMO*_*GS-OX2~4*_, all homologs of glucosinolate synthesis genes in *A. thaliana* are found in *Brassica rapa*[39]. However, we found homologs of *AOP3*, *CYP79F2*, *MYB76* and *FMO*_*GS-OX2~4*_ in the transcriptome of *C. sativa* (data not shown), again confirming the close relationship between this species and *A. thaliana*.

## Conclusions

Using the Illumina platform, we derived by paired-end and *de novo* technology a dataset comprising 83,493 unigenes from the *Camelina sativa* transcriptome. Of these, 67,791 were annotated with gene descriptions from NR, Swiss-Prot, COG and KEGG databases. After removal of redundancies, 25,329 non-redundant sequences were obtained. We discovered differences between KEGG pathways of *A. thaliana* and *C. sativa* (Additional files 4 and 6). Though both of these species belong to the Brassicaceae and share a close genetic relationship, there was inter-specific variation in genes involved in some metabolic pathways. This is a first report of *Camelina sativa* transcriptome analysis; it provides a valuable database for further research on this species.

## Methods

### RNA preparation from plant materials

*Camelina sativa* ecotype MT-5 was obtained from Dr. Alice Pilgeram, Montana State University, USA. We grew plants in a greenhouse under normal sunlight in Hong Kong, China. Both young and mature leaves were collected for RNA extraction. Leaves we collected were immediately frozen in liquid nitrogen and stored at −80°C until further use. For Illumina sequencing, total RNA was extracted from pooled leaves and then digested with DNase I following manufacturer’s instructions (RNeasy Plant Mini Kit, Qiagen, Hong Kong). RNA was quantified with an Agilent 2100 Bioanalyzer RNA Nanochip. At least 20 μg of total RNA at a concentration of ≥ 400 ng/μl, OD260/280=1.8~2.2, RNA 28S:18S ≥ 1.0, and RNA Integrity Number (RIN) ≥ 7.0 were used for cDNA library preparation.

### Preparation of cDNA library for transcriptome sequencing

Illumina sequencing was performed at the Beijing Genomic Institute, Shenzhen, China following manufacturer’s instructions. First, poly-T oligo-attached magnetic beads (Illumina) were used to isolate poly (A)^+^ RNA from the total RNA quantified. Subsequently, poly (A)^+^ RNA was purified and fragmented into smaller pieces (200–700 nt) using divalent cations at 94°C for exactly 5 minutes. First-strand cDNA was synthesized with SuperScript II reverse transcriptase and random primers using the small fragment RNAs as templates. Second-strand cDNA was then synthesized using GEX second strand buffer, dNTPs, RNase H and DNA polymerase I. These cDNA fragments were further processed with end repair and phosphorylation using T4 DNA polymerase, Klenow DNA polymerase, and T4 polynucleotide kinase. The repaired cDNA fragments were 3’ adenylated using Klenow Exo-; Illumina’s paired-end adapters were ligated to the ends of these 3’-adenylated cDNA fragments. The products from this ligation reaction were electrophoresed on a 2% (w/v) TAE-agarose gel and purified to select templates of different sizes for downstream enrichments. cDNA fragments of 200 bp (±25 bp) were excised from the gel. To enrich the quantity of the cDNA template, fifteen cycles of PCR amplification were performed using PCR primers PE1.0 and PE2.0 with Phusion DNA Polymerase. Finally, a cDNA library was constructed with a fragment length range of 200 bp (±25 bp).

### Data filtering and *de novo* assembly by SOAPdenovo

The raw “reads” were filtered to obtain high quality *de novo* transcriptome sequence data. First, all reads with adaptor contamination were discarded. Second, reads with unknown nucleotides comprising more than 5% were removed. Third, low-quality reads with ambiguous sequence “N” were discarded. Subsequently, *de novo* assembly of the clean reads was performed using SOAPdenovo, which first combined reads with selected overlap lengths to form longer fragments without N; these fragments are termed contigs. The reads with overlapping regions were then mapped back to the contigs for scaffold construction; with paired-end reads it is possible to detect contigs from the same transcript and the distances between them. In the next step, SOAPdenovo connected the contigs using N to represent unknown sequences between each pair of contigs to produce scaffolds. Paired-end reads were used again to fill the gaps in the scaffolds and get sequences with the fewest Ns that could be extended from either end. These sequences are termed unigenes. Finally, sequences of assembled unigenes were translated into amino acids in order to run BLASTX at an E-value threshold of 1.0E-5 following priorities of the non-redundant protein (NR) database (http://www.ncbi.nlm.nih.gov), the Swiss-Prot protein database (http://www.expasy.ch/sprot), the Kyoto Encyclopedia of Genes and Genomes (KEGG) pathway database (http://www.genome.jp/kegg) and the Cluster of Orthologous Groups (COG) database (http://www.ncbi.nlm.nih.gov/COG). The best aligning result was used to decide the sequence direction of each unigene. If a unigene had no alignment hit (match) in the above databases, ESTScan software [16] was used to predict the CDS domain and its direction. For unigenes with sequence direction, sequences from the 5’ end to the 3’ end were provided. For those without sequence direction, we obtained sequences from assembly software. TIGR Gene Indices Clustering Tools (TGICL) software [40] was used to clusters scaffolds to produce unigenes with the fewest redundant sequences.

### *de novo* assembly by Trinity

In order to verify transcriptome assembly by SOAPdenovo, we ran Trinity [13] to assemble clean reads into transcripts. Trinity focuses more on splice isoforms. It first forms contigs that represent the significant parts of individual isoforms. Then, it clusters the contigs into individual groups such that contigs from isoforms likely to be from the same gene are grouped together. Lastly, each group of contigs is processed separately. Furthermore, Trinity is based on paired-end reads and attempts to reconstruct transcripts for splice isoforms in each group. Different from SOAPdenovo, Trinity did not fill gaps with ‘N’s to represent unknown sequences when two contigs were connected. Instead, Trinity built a K-mer dictionary from all clean reads based on frequency and considered each K-mer as an initial contig. Each initial contig (in descending frequency order) was extended by selecting the most frequent K-mer in the dictionary with K-1 overlaps with the current contig end, until neither direction could be extended further. Subsequently, contigs were pooled if they shared at least one K-1-mer and there were reads across the junction sites. A de Brujin graph was constructed for each contig pool. Finally, each de Brujin graph was compacted and linear sequences representing each alternative splicing form and/or high similar transcripts were produced.

### Unigene annotation and classification

The annotation of unigenes was performed using various bioinformatics procedures. Unigenes were firstly translated into amino acids in six frames and aligned with BLASTX to protein databases including NR, Swiss-Prot, KEGG and COG (E-value ≤ 1.0E-5); the protein with highest sequence similarity was retrieved and annotated to each unigene. With NR annotation, Blast2GO [41] software was used to get GO annotation defined by molecular function, cellular component and biological process ontologies. After obtaining GO annotation for every unigene, WEGO software [42] was used to produce GO functional classification for all unigenes and to interpret the distribution of species’ gene functions at the macro level. Unigene sequences were also aligned to the COG database to predict and classify possible functions. Pathway assignments were determined following the Kyoto Encyclopedia of Genes and Genomes pathway database using BLASTX with an E-value threshold of 1.0E-5.

## Competing interests

The authors declare that they have no competing interests.

## Authors’ contributions

BLL: designed the experimental plan. CL: carried out the experiments, analyzed the data and wrote the manuscript. XL: carried out Trinity assembly analysis. SMY: verified SOAPdenovo assembly results by Trinity and revised the manuscript. All authors read and approved the final manuscript.

## Supplementary Material

Additional file 1 **Supplementary Figures for this study. Figure S1** Overview of assembly by SOAPdenovo. (**a**) Length frequency distribution of contigs obtained from *de novo* assembly of high-quality clean “reads”. (**b**) Length frequency distribution of gap ratios (N/size) in assembled scaffolds. (**c**) Frequency distribution of assembled scaffold lengths. (**d**) Length frequency distribution of unigenes produced by contig joining, gap filling, and scaffold clustering. (**e**) Gap frequency distribution of assembled unigenes. x-axis values are ratios of gap length to length of assembled unigenes. y-axis values are frequencies of unigenes containing gaps. (**f**) Random frequency distribution of Illumina sequencing reads in assembled unigenes. x-axis values are relative positions of sequencing reads in assembled unigenes. The orientation of unigenes is from the 5’ end to the 3’ end. **Figure S2** Venn Diagrams of the three categories of GO. In total, 33,475 unigenes were assigned to at least one GO category. **Figure S3** Venn diagram results from diverse databases. (**a**) Venn diagram showing the number of unigenes matched to sequences in NR, Swiss-Prot and KEGG databases. All annotations were based on best BLASTX hits with E-Values ≤ 1.0E-5. The overlapping regions represent the number of unigenes that matched in different databases. (**b**) Venn diagram showing the number of unigenes in NR, Swiss-Prot, KEGG and COG databases. All annotations were based on the best BLASTX hits with E-Values ≤ 1.0E-5. **Figure S4***Camelina sativa* transcriptome coding sequence (CDS) predicted by BLASTX and ESTScan software. (**a**) Number of predicted CDS with gap ratio frequency distribution (N/size). (**b**) Length frequency distribution of predicted CDS. (**c**) Length frequency distribution of predicted protein sequences. (**d**) Gap ratio frequency distribution of CDS predicted by ESTScan software. (**e**) Length frequency distribution of CDS predicted by ESTScan software. (**f**) Length frequency distribution of protein sequences predicted by ESTScan software. Click here for file

Additional file 2 **NR annotations of *Camelina sativa *with an E-value threshold of 1.0E-5.** Click here for file

Additional file 3 **722 unigenes annotated to *Brassica *and 190 annotated to *Brassica *disease resistance protein coding genes.** Click here for file

Additional file 4 119 KEGG pathways with pathway ID and KO information. Click here for file

Additional file 5 **Number of annotated unigenes involved in fatty acid synthesis in *Camelina sativa.*** Click here for file

Additional file 6 **Differences in 119 KEGG pathways between *Camelina sativa *and *Arabidopsis thaliana.*** Click here for file
