# Supplementary material for: De novo assembly and characterization of Camelina sativa transcriptome by paired-end sequencing
Source: BMC Genomics. 2013 Mar 5;14:146. doi: 10.1186/1471-2164-14-146 (PMC3635884; doi:10.1186/1471-2164-14-146)
Supplement: Additional file 1 — Supplementary Figures for this study. Figure S1 Overview of assembly by SOAPdenovo. (a) Length frequency distribution of contigs obtained from de novo assembly of high-quality clean “reads”. (b) Length frequency distribution of gap ratios (N/size) in assembled scaffolds. (c) Frequency distribution of assembled scaffold lengths. (d) Length frequency distribution of unigenes produced by contig joining, gap filling, and scaffold clustering. (e) Gap frequency distribution of assembled unigenes. x-axis values are ratios of gap length to length of assembled unigenes. y-axis values are frequencies of unigenes containing gaps. (f) Random frequency distribution of Illumina sequencing reads in assembled unigenes. x-axis values are relative positions of sequencing reads in assembled unigenes. The orientation of unigenes is from the 5’ end to the 3’ end. Figure S2 Venn Diagrams of the three categories of GO. In total, 33,475 unigenes were assigned to at least one GO category. Figure S3 Venn diagram results from diverse databases. (a) Venn diagram showing the number of unigenes matched to sequences in NR, Swiss-Prot and KEGG databases. All annotations were based on best BLASTX hits with E-Values ≤ 1.0E-5. The overlapping regions represent the number of unigenes that matched in different databases. (b) Venn diagram showing the number of unigenes in NR, Swiss-Prot, KEGG and COG databases. All annotations were based on the best BLASTX hits with E-Values ≤ 1.0E-5. Figure S4Camelina sativa transcriptome coding sequence (CDS) predicted by BLASTX and ESTScan software. (a) Number of predicted CDS with gap ratio frequency distribution (N/size). (b) Length frequency distribution of predicted CDS. (c) Length frequency distribution of predicted protein sequences. (d) Gap ratio frequency distribution of CDS predicted by ESTScan software. (e) Length frequency distribution of CDS predicted by ESTScan software. (f) Length frequency distribution of protein sequences predicted by ESTScan software [file 1471-2164-14-146-S1.docx]

**Additional file 1: Supplementary Figures in this study.**


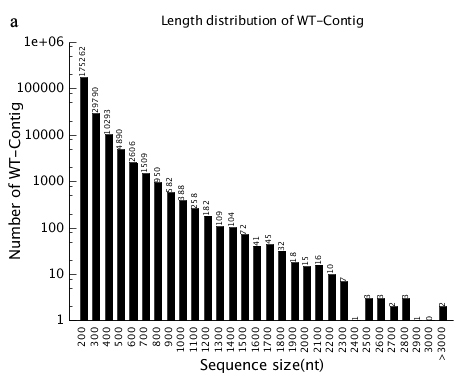

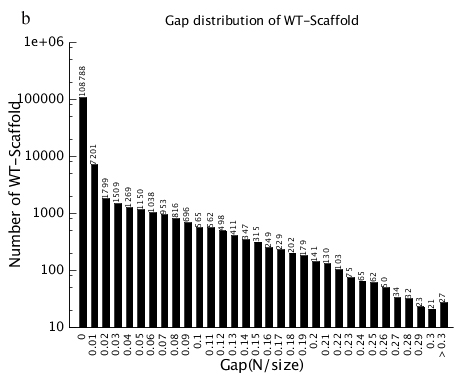


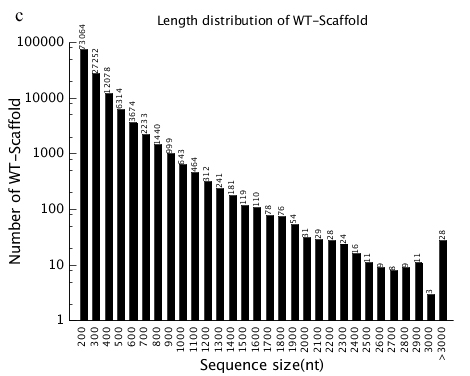

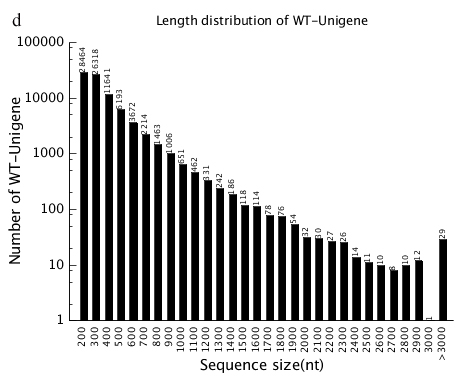


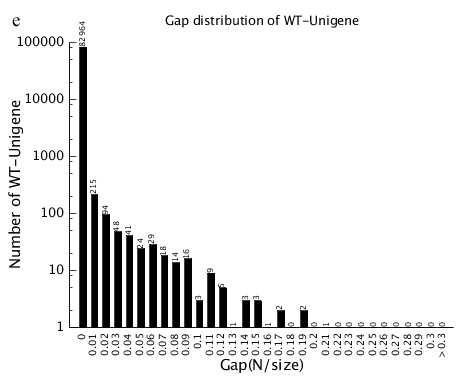

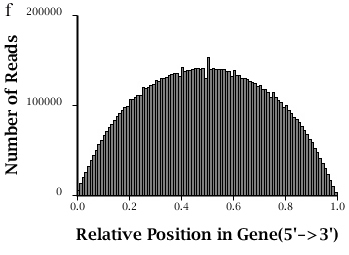


**Figure S1 Overview of assembly by SOAPdenovo.** (a) Length frequency distribution of contigs obtained from *de novo* assembly of high-quality clean “reads”. (b) Length frequency distribution of gap ratios (N/size) in assembled scaffolds. (c) Frequency distribution of assembled scaffold lengths. (d) Length frequency distribution of unigenes produced by contig joining, gap filling, and scaffold clustering. (e) Gap frequency distribution of assembled unigenes. x-axis values are ratios of gap length to length of assembled unigenes. y-axis values are frequencies of unigenes containing gaps. (f) Random frequency distribution of Illumina sequencing reads in assembled unigenes. x-axis values are relative positions of sequencing reads in assembled unigenes. The orientation of unigenes is from the 5' end to the 3' end..

**
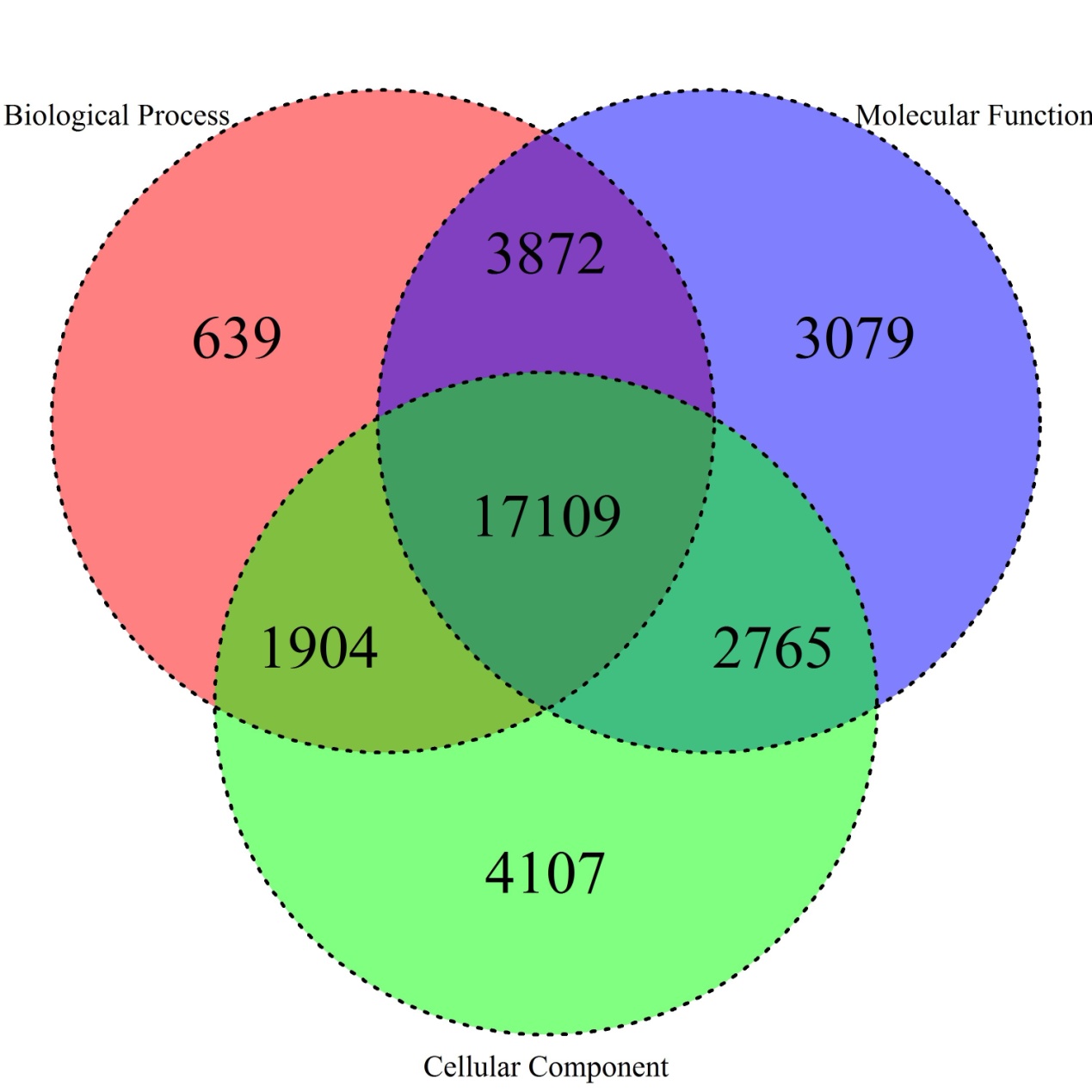
**

**Figure S2 Venn Diagrams of the three categories of GO.** In total, 33,475 unigenes were assigned to at least one GO category.


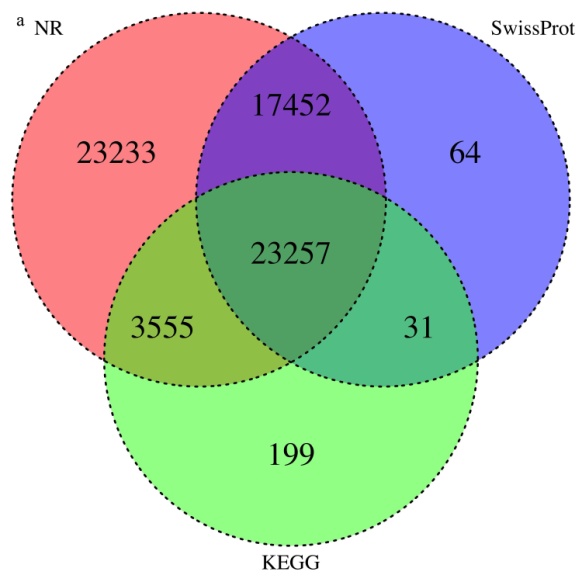

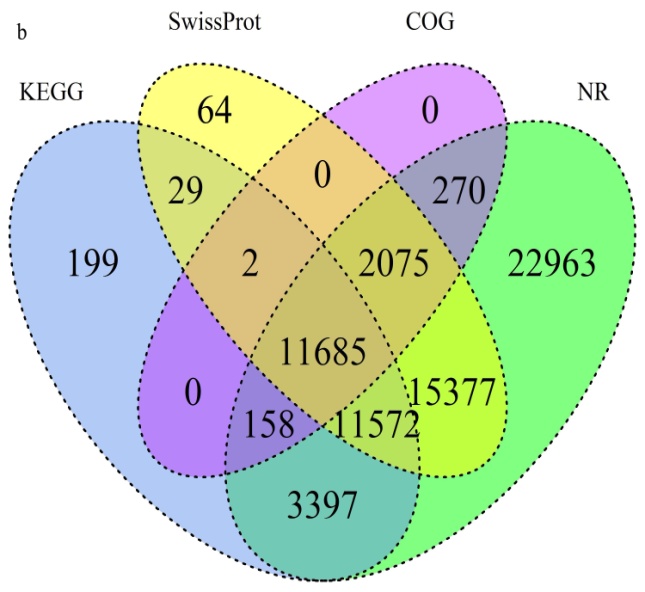


**Figure S3 Venn diagram results from diverse databases.** (a)Venn diagram showing the number of unigenes matched to sequences in NR, Swiss-Prot and KEGG databases. All annotations were based on best BLASTX hits with E-Values ≤ 1.0E-5. The overlapping regions represent the number of unigenes that matched in different databases. (b) Venn diagram showing the number of unigenes matched to sequences in NR, Swiss-Prot, KEGG and COG databases. All annotations were based on the best BLASTX hits with an E-Values ≤ 1.0E-5. The overlapping regions represent the number of unigenes that matched in different databases.


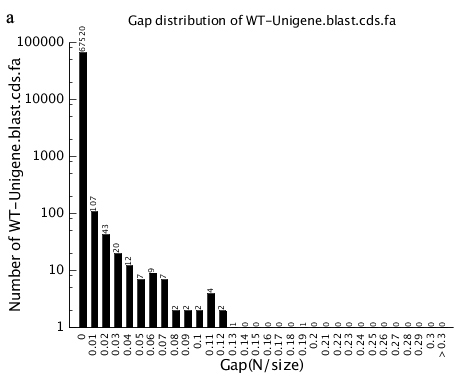

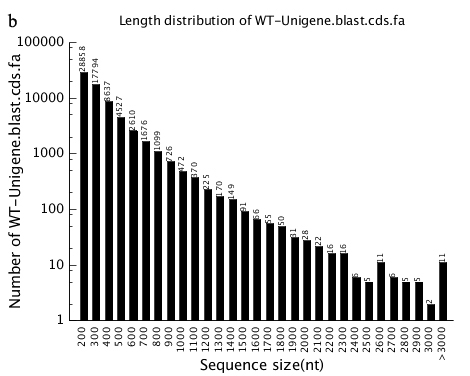


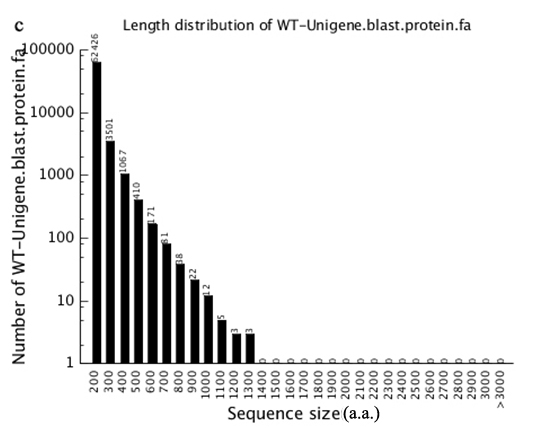

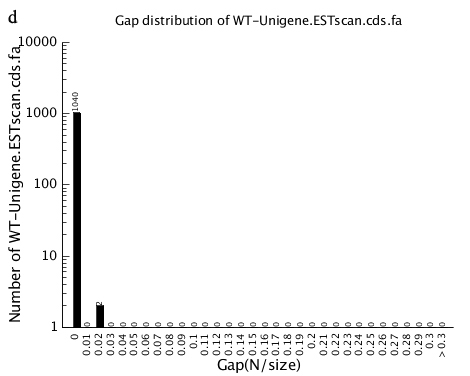


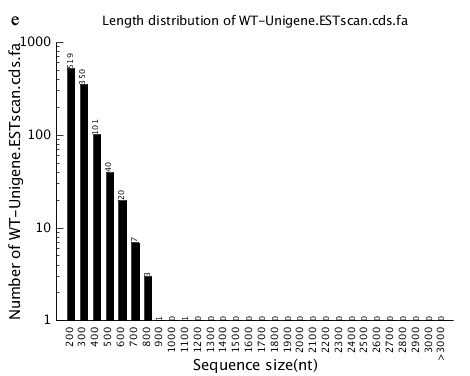

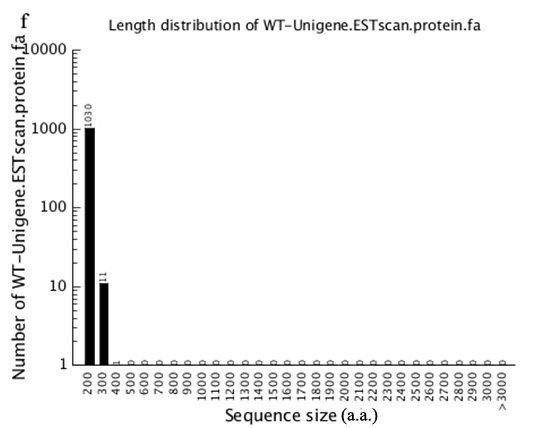


**Figure S4** ***Camelina sativa* transcriptome coding sequence (CDS) predicted by BLASTX and ESTScan software.** (a) Number of predicted CDS with gap ratio frequency distribution (N/size). (b) Length frequency distribution of predicted CDS. (c) Length frequency distribution of predicted protein sequences. (d) Gap ratio frequency distribution of CDS predicted by ESTScan software. (e) Length frequency distribution of CDS predicted by ESTScan software. (f) Length frequency distribution of protein sequences predicted by ESTScan software.
